# Low back pain under control - Bartzokas' maneuver: a case report

**DOI:** 10.4076/1757-1626-2-7161

**Published:** 2009-07-07

**Authors:** Konstantinos Chatzinikolaou, Theoharis Paschalis, Aristides Bartzokas, Ioannis DK Dimoliatis

**Affiliations:** 1 University of Ioannina School of Medicine, Department of Hygiene & Epidemiology, University Campus45110, IoanninaGreece; 2 University of Ioannina, Department of Physics, University Campus45110, IoanninaGreece

## Abstract

Low back pain (lumbago) is a common health problem globally. It is related to age, modern lifestyle (no exercise etc), and injuries. Its treatment includes a very broad spectrum of methods and its prevention is still unclear.

We present Aristides, a 52-year old male, who has been suffering from low back pain for 10 years, and discovered a preventive and therapeutic maneuver that has helped him to significantly decrease the acute and chronic attacks of low back pain that were haunting him. We present the “Bartzokas' maneuver”, a very simple and effective therapeutic - as well as preventive - manipulation with no known adverse effects.

## Introduction

Low-back pain is a major health problem among western industrialized countries, and a major cause of medical expenses, absenteeism and disablement; it is the second most frequent pathologic situation after the common cold; it has been calculated that 65-80% of the general population has at least one episode of low back pain during their lives [1].

People with acute low-back pain usually experience improvements in pain, disability, and return to work within one month, further but smaller improvements occur up to three months, after which, pain and disability levels remain almost constant and most people will have at least one recurrence within 12 months.

As people age, bone strength, muscle elasticity and tone tend to decrease, the discs begin to lose fluid and flexibility, which decreases their ability to cushion the vertebrae. Most low back pain cases are due to minor or major injuries of the connective elements of the lumbar segment of the vertebral column that originate either from compression after lifting a heavy object or from excessive stretching. Bulging disc, sciatica, spinal degeneration, spinal stenosis, osteoporosis, skeletal irregularities, fibromyalgia, spondylitis can be associated with low back pain. Pregnant women can suffer from low back pain and also people without a good physique even from an incorrect sleeping position [2].

Low back pain can be acute or chronic. In the case of acute pain the cause is usually injury of the soft tissue, like ligaments or muscles. In the case of chronic pain the causes can also be physical, like osteoarthritis, rheumatoid arthritis, intervertebral disc problems [3].

There are two basic ways of dealing with the problem, surgical and non-surgical. An operation takes place mostly when all the other treatments were ineffective and the patient develops neurological problems in addition to the pain, which usually happens with hernias of the intervertebral discs, spinal stenosis, spinal injuries, spinal hematoma, scoliosis etc [4].

This case report presents a new maneuver preventing and curing instantly low back pain attacks, discovered by a patient who did not stay a passive observer of his illness.

## Case presentation

Aristides Bartzokas, 52, a Caucasian male, 1.77 m high, and weighing 79 Kgs (BMI 25.2), is currently working as an Associate Professor of Meteorology and Climatology in the University of Ioannina, Greece. He is married and has two children. He used to smoke 1-2 cigarettes a day, since he was 20 years old until 5 years ago when he gave up smoking. He regularly consumes alcohol, though in small amounts (1-2 glasses of wine every day). He has an allergy called seborrheic dermatitis which is treated with betamethasone 17 - valerate 15g that contains no cortisone. He had a stone in his right kidney in 1980 (24 years old) that was eliminated with urination. Even though Aristides spends a lot of his time sitting due to his work, he is also an active person since he goes mountaineering. His brother was diagnosed with hernia of the intervertebral disc after lifting weight, had low back pain and had to go through disc resection.

Ten years ago, while doing some chores, he lifted a heavy object, and since then he suffers from low back pain. Within these past ten years, three times the problem was very intense due to acute low back pain (he would grade his pain with 9 on a scale from 0 to 10) and Aristides had to stay in bed in order to deal with it. He was diagnosed with low back pain on the first visit at the orthopaedist. The X-ray examination also revealed that he had spinal bifida, but that was considered not responsible for his pain.

Since then Aristides began to adjust to his condition, discovering new techniques in order to deal with it. However, he never used a corset or any medication (he used anti-inflammatory tablets only once). Even when he stays in bed he only rests or he is given massage.

**Figure 1.**
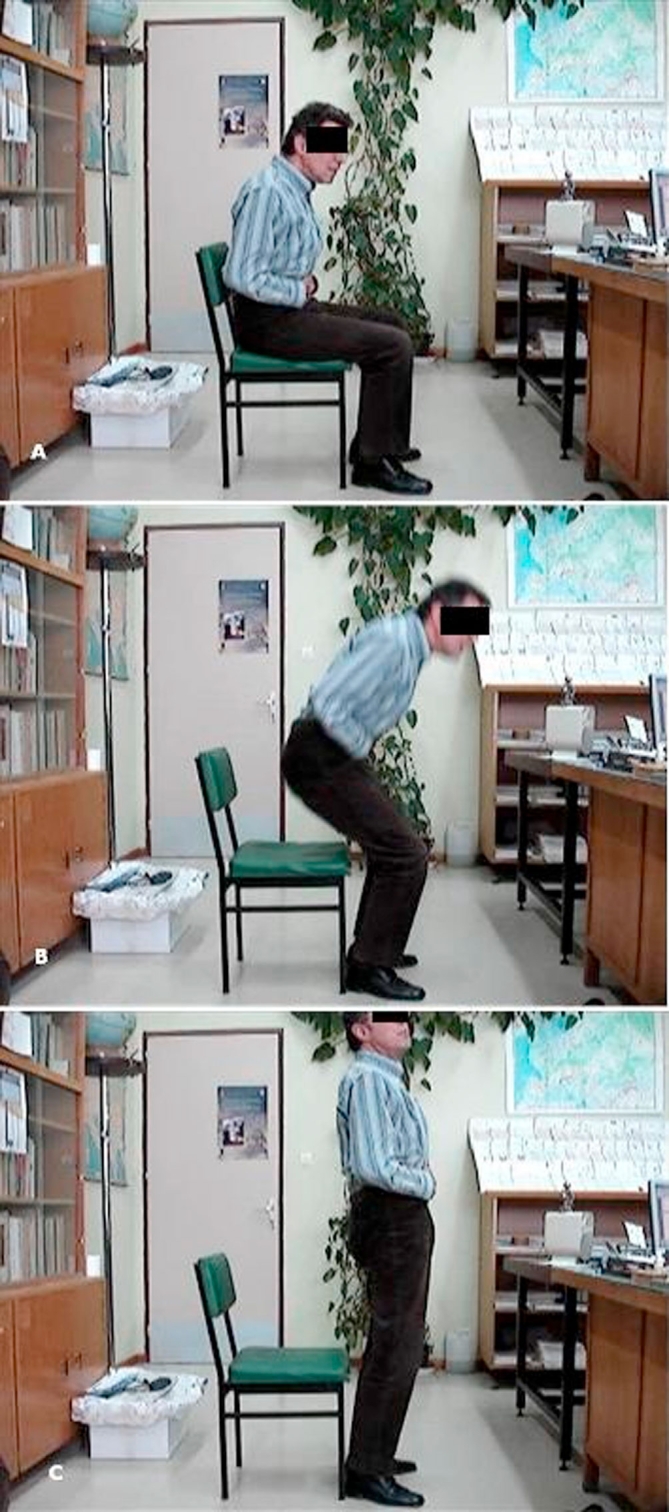
The “Bartzokas' maneuver”. **Top:** while seated, ‘sucks in’ the abdomen and simultaneously presses his abdomen with his hands. **Middle:** elevation; the alignment of the spinal column is characteristic, especially of the lumbar segment. **Below:** completion of the elevation to standing position, with alignment of the femurs and knees. During the whole elevation the spinal column is ‘maintained’ still and aligned. (Photo: Konstantinos Chatzinikolaou).

Aristides reports that when his waist aches, simple everyday movements, like getting up from bending, getting out of bed, getting up from a chair and getting out of the car, become very difficult. He also reports that, sometimes, the pain becomes very intense when he tries to choke his sneezing. While trying to avoid the pain and the acute crisis, he discovered by himself, without the advice of any physicians, an effective way that helps him prevent or cure the acute episodes. As you can see in the photos (Figure 1) and the video (supplementary file), when Aristides is sitting down and wants to get up, he sucks in his abdomen and in more difficult occasions he presses his abdominal muscles with his hands and then gets up. He practices this technique every day, whenever he needs to get up and, mainly, when he feels that his low back is starting to ache. This way he succeeded in preventing reducing the acute crises and the occasional low back pain that immobilizes him.

Aristides remembers an incident very well; he was waiting in a queue for a long time. While coming out and without stumbling - the ground was even - an acute crisis began and he fell on the ground (bended) from the pain. In order to get up, he had to hold the fence along the pavement and pressed his abdomen with the technique he discovered. He then tried to walk to the next bus stop but it was impossible; he managed only when he kept his abdomen pressed as much as possible, by “sucking” his abdominal muscles and by pressing them with his hands, curing instantly his acute crisis without any artificial or medical help.

He reports two more postures that relieve his pain: in bed, he lies down on the side “foetus posture”; and, when he is working sitting on a chair for a long time, he lifts his body by supporting his hands on the chair, while his legs stay numb.

## Discussion

Which is the possible mechanism of action of the “Bartzokas' maneuver”? If we take a good look at his maneuver, we can see that he presses his abdomen with his hands. More precisely, he presses the main muscles of the anterolateral wall which are the external oblique, the internal oblique, transversus abdominis and rectus abdominis. Anatomically, these muscles are inserted on the anterior part of the pubic bone. By pressing them with his hands, Aristides succeeds in making a small bent of the pelvis backwards that as a result aligns and stabilizes the lumbar boss of the vertebras at the moment of getting up. The stabilization of the spinal column is very important because it prevents the bend of the vertebras by preventing further discomfort of a possible hernia of the intervertebral disc or even further dilatation of the interaccessory muscles that are always disturbed and are in spasms during the acute crisis [5].

We can compare Bartzokas' maneuver with pelvic belt which practices a continuous supportive pressure on muscles and ligaments of the pelvic area including the abdominal muscles, something that prevents and relieves low back pain [6]. In our case Bartzokas' maneuver is something more than a pelvic belt considering that it can be applied anytime, anywhere, not only to prevent but also to cure acute low back pain and it is cost effective. Attention, Bartzokas' maneuver was discovered by the patient himself and is a genuine action in order to prevent and cure low back pain crisis.

### Patient's perspective

#### Living with low back pain (lumbago)

You get out of bed in the morning and you have difficulties in getting up, you go wash and you ache, or you try to get out of the car and you are careful because you are scared that ‘if I do a sudden move I will not be able to move’. You are talking about an almost everyday problem, that is, if I try to get out of bed incautiously, I might, indeed, become stiff and that will make me fall down, and I will have to bend. The problem is the stiffness, you try to get up and you say “oh, I can't; my back” and you sit back again.

#### The discovery of the maneuver

Once, just by chance, I had noticed that if my abdomen is sucked in, I get up as without any problems, as if I'm teenager of eighteen years. If the abdomen is out, it hurts, if it is a difficult case of pain. Therefore, I put my hands and I push it myself. This way I give additional support and then I get up comfortably. I tried it again and again and it worked every single time! I discovered the solution of my problem.

#### Cure and prevention

I remember once I had to go to the Institute of Social Insurance to get my late father's papers and it got me and I fell down. I couldn't get up. In order to get up, I had to hold the fence along the pavement and sucked my abdomen in and got up. Then, I realised that I could not move my leg in order to take a step. I did it by further pushing the abdomen by using my hands. It took me five minutes, and then I could walk almost freely. If it is a difficult day, because, for example, the previous I was carrying loads, the next few days I do this maneuver every time I try to get up, 'cause I know that as soon as I try to get up without this I get the pain, and therefore I need to prevent it from happening. It sort of warns you a bit. It is like the bear that is restricted at the zoo in a place that is surrounded by a thin wire with high voltage; it knows that it will be electrocuted if it touches it. This happens to me too, I am afraid; therefore, I take measures myself.

## Conclusion

This case report presents a maneuver unknown to bibliography which works preventively and therapeutically that may help a lot of patients to avoid the symptoms of low back pain, thus improving their quality of life. It is remarkable that the manipulation was discovered by the patient himself who has no connection whatsoever with medicine but the need for relief and prevention drove him to the discovery of an effective maneuver which is easy to be applied from anyone and anywhere with direct results and with no side-effects. Further research is needed to clarify to which patients with low back pain it is effective; however, we feel any low back pain patient should be taught Bartzokas' maneuver: perhaps s/he could solve his/her problem.

## Supplementary Files

Video 1. The “Bartzokas' maneuver”. The video (MP4 - 2.1 MB) can be accessed here: http://casesjournal.com/casesjournal/article/downloadSuppFile/7161/19300 He (Aristides Bartzokas) is saying: “… you can suck your abdomen in - and if you cannot do it very much you can help with your hands - and you make this movement and raise yourself very easily. Without any pain.” (Video: Konstantinos Chatzinikolaou).
